# Examining the Relationship between Workplace Fun and Innovative Behavior among Nurses: The Mediating Effect of Innovation Support and Affective Commitment

**DOI:** 10.1155/2024/9629172

**Published:** 2024-07-31

**Authors:** Maryam Hashemian, Azam Hashemian Moghadam, Mirtohid Hosseini, Islam Azizpour, Alireza Mirzaei

**Affiliations:** ^1^ Students Research Committee School of Nursing and Midwifery Ardabil University of Medical Sciences, Ardabil, Iran; ^2^ Department of Psychology Faculty of Education and Psychology Ferdowsi University of Mashhad, Mashhad, Iran; ^3^ Department of Critical Care Nursing School of Nursing and Midwifery Guilan University of Medical Sciences, Rasht, Iran; ^4^ Department of Emergency Nursing School of Nursing and Midwifery Ardabil University of Medical Sciences, Ardabil, Iran

## Abstract

**Aim:**

This study investigated the role of cultural, organizational, and managerial support, workplace fun, affective commitment, innovative behavior with innovative output, and also the mediating role of innovative behavior in the framework of a causal model.

**Background:**

Innovation is the driving force of development in hospitals, and the quality of healthcare is closely related to hospital innovation. Today, nurses with innovative behaviors are the biggest asset of any hospital because they are involved in any improvement and progress.

**Methods:**

This descriptive cross-sectional correlational study was conducted using causal modeling methods, including path analysis and structural equation modeling. Using the proportional stratified sampling method, 321 nurses from Ardabil teaching hospitals were included in the study. Data were collected by standard demographic characteristics, innovative behaviors, innovative support, workplace fun, and affective commitment questionnaires. Partial least square structural equation modeling (PLS-SEM) was used to test the conceptual model using PLS-SMART 2 software.

**Results:**

Cultural support had a positive and significant effect on innovative behavior by affecting organizational support and then managerial support. In addition, workplace fun had a positive and significant effect on innovative behavior directly and indirectly through the mediating role of affective commitment. Finally, innovative behavior also had a positive and significant effect on nurses' innovative output.

**Conclusion:**

Supervisors and managers can adopt the organizational and managerial support approach to improve the nurses' innovative behaviors. Workplace fun will also improve nurses' innovative behaviors and affective commitment, thereby increasing their innovative output. *Implications for Nursing Management.* By adopting organizational and managerial support for nurses' innovative behaviors, managers should take measures that promote workplace fun and affective commitment to improve nurses' innovative output by encouraging innovative behaviors.

## 1. Introduction

To provide quality healthcare, hospitals must adopt innovative practices [1]. With the constantly evolving health technology and increasing demand for healthcare services, hospitals need to improve their efficiency, creative thinking, and value [2]. Innovative behavior involves generating, cultivating, and implementing new ideas within an organization or workgroup [3]. Implementing innovative nursing practices in hospitals directly influences their performance [1, 4]. The particular area where a hospital implements innovative nursing practices has a significant impact on its overall performance [1, 4]. Continuous innovation of nurses not only fulfills their developmental needs but helps managers improve their work performance [1]. One strategy that managers can employ to encourage innovation is to create a fun work environment [4].

Workplace fun includes socializing with colleagues, celebrating at work, personal freedoms, participating in challenges, performance-based competitions, and generally fun activities at work [5]. Workplace fun affects the attitudes and working behaviors of employees and strengthens their creativity of employees. The effects of workplace fun on innovation have been mentioned in studies [1]. Tews et al. reported that a fun work environment is more attractive to employees than their salary and promotion [6].

Employees usually pursue their affective needs after meeting material needs [7]. Affective commitment describes the employee's emotional dependence on identification and participation in the organization and the individual's emotional orientation towards the organization. Affective commitment is revealed when employees accept that initiative is necessary to maintain the image and reputation of the organization, promote the organization, work hard for the development of the organization, and have high trust and loyalty to the organization [1]. In addition, the role of organizational commitment in the emergence of innovative behaviors has also been seen [8]. For people's innovative behaviors to be transformed into new and effective products and methods, more efforts of employees are needed, and the support of managers and some influential organizational variables are also required [3]. Different leadership styles affect organizational innovation [7].

The more supportive the leadership style, such as transformational leadership, the more innovative behaviors will be in that organization. In the studies conducted on nurses, the cultural climate supporting innovation in the organization and managerial support for innovation has had a positive relationship with the innovative behaviors of nurses [9]. Despite the abovementioned cases, employees have often shown that their managers still deny them from having a fun workplace [10].

The effect of a supportive work environment on employee innovation is significant, but it is often neglected in Iranian hospitals. Despite the importance of recreational activities in promoting innovative behaviors, more research needs to be done in this field, especially among nurses in Iran. To fill this gap, a pioneering study was conducted in Ardabil City, which examined the relationship between innovative behavior, workplace fun, support for innovation, and affective commitment among nurses. These findings illuminate the benefits of a positive work culture and inform future efforts to foster innovation in healthcare settings.

## 2. Literature Review

Numerous factors can affect innovation, including individual and contextual factors. Three factors that influence employees' innovation are cultural, managerial, and organizational support [3, 11]. Among these factors, some, such as the direct manager's influence, support, and leadership style, can have more significant effects than cultural factors. Innovation is a managerial process that requires approval from the organization and its managers [12]. However, the national culture governing the society can affect the leadership style of managers and the organizational support provided [3]. According to Sönmez et al.'s study [4], leadership support has a positive impact on increasing innovative behaviors and ultimately leads to an increase in the innovative output of employees. In Yang et al.'s study [13], it was found that humble leadership fostered innovative behaviors in nurses. Based on these findings, we hypothesized that innovative support positively correlates with innovative behaviors.

Over the past few years, the importance of having a fun work environment and its impact on employee behavior and performance has been recognized [14]. Creating a pleasant and humorous atmosphere in the workplace encourages positive behavior among employees [15]. A fun-filled workplace can lead to a reduction in stress levels and increase enjoyment, resulting in better engagement with the team, colleagues, and the organization as a whole. In addition, having fun at work has a positive effect on creativity, work performance, and the affective commitment of employees [16]. By incorporating fun activities into the work environment, employees can show more innovative behaviors and improve the overall work environment [1]. Managers offer their care and support for employees by creating exciting conditions in the work environment, and employees show more organizational behaviors, such as innovation, to return it to managers [17]. The results of Jing et al.'s study [1] showed that nurses who had fun in the work environment showed more innovative behaviors, and this mechanism could be due to the effects of affective commitment created in employees seeking fun in their work environment. When the organization creates a calm environment through entertainment for nurses, they will be more active and creative in terms of thinking [1]. Therefore, the following hypothesis was developed: workplace fun positively correlates with innovative behaviors.

Affective commitment is the psychological identification of employees with the goals and values of the organization and the level of belief and support for it [18]. Affective commitment is revealed when employees take the initiative to maintain the image and reputation of the organization, inform the public about the organization, strive for the development of the organization, and have high trust and loyalty to the organization [1, 15]. Such people show more ability to participate in the activities of an organization. They are always ready to try beyond their duty to achieve the organization's goals [12, 18]. Affective commitment can predict employees' feelings, such as trust and stress, or organizational behaviors, such as absenteeism from the workplace or organizational innovation [19]. It has been observed that employees with a higher level of commitment to their organization continually develop creative solutions to work-based problems and, thus, show a greater tendency to engage in innovative behaviors [20]. Therefore, we hypothesized that affective commitment positively correlates with employees' innovative behaviors.

One of the most critical issues that define a person's behavior in an organization is affective commitment. Affective commitment shows the individual's identity and participation and enjoyment in the organization. People who have an affective commitment to the organization go beyond simply staying in the organization. One of the factors that can create affective commitment in a person is fun at work. Putri et al.'s study [19] showed that fun at work through employees' work participation can lead to affective commitment in employees. Also, Al Bazzal's study [21] also showed that fun in the work environment directly leads to affective commitment in people. Other studies show that having fun at the workplace by creating a happy atmosphere for employees leads to the creation of teamwork spirit and thus leads to an increase in affective commitment in the individual [22]. The existence of such arguments led us to create the following hypothesis: workplace fun has a positive relationship with affective commitment.

There have been few studies conducted to examine the relationship between innovative behavior and workplace fun, affective commitment, and support for innovation. To fill this gap in research, it is important to study all the influential variables proposed in the structural model of innovation in the hospital and the treatment environment. This includes examining the importance of these variables and refining the relationships between them through the inclusion of other structures such as workplace recreation and affective commitment. By doing so, we can gain a more accurate theoretical explanation of the behavior and ultimately the innovative output of nurses in the workplace.

**Figure 1:**
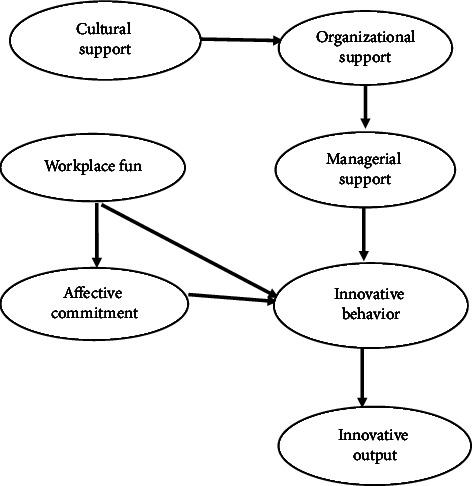
A graphical model highlighting the hypothesized relationships.

Therefore, the purpose of this study is to examine the indirect role of the hierarchies of cultural, organizational, and managerial support, as well as the direct and indirect effects of workplace entertainment, through the mediation of affective commitment. This study aims to examine the relationship between innovative behavior and the output variable, while also exploring the potential mediating role of affective commitment and managerial and organizational support on innovative behavior. The study will focus on nurses and test a conceptual model using the structural equation modeling method. The model was developed based on existing theoretical and research background. The study hypotheses are displayed in Figure 1.

## 3. Methods

### 3.1. Aims

Although there are studies related to the issues raised above, the effect of workplace fun on the innovative behavior of nurses in the working environment of Iranian hospitals is a topic that has not been sufficiently researched and its mechanisms have not been revealed well. Therefore, this study is the first to investigate the relationship between innovative behavior and workplace fun, innovation support, and affective commitment among nurses in Ardabil City, Iran.

### 3.2. Design

This descriptive cross-sectional research was conducted using causal modeling methods, including path analysis and structural equation modeling.

### 3.3. Participants

The statistical population included all nurses (*n* = 1284) of five teaching hospitals in Ardabil, a city located in the northwest of Iran. The inclusion criteria were nurses who worked in clinical settings and had at least 6 months of nursing experience. They also reported that they had no history of physical or mental illnesses. Nurses who had continuous leave of absence for more than three months and nursing supervisors and managers were excluded from the study.

### 3.4. Data Collection

A study was conducted on 291 nurses to determine an appropriate sample size using Krejcie and Morgan's [23] table. This method is used when the entire study population is known. For studies using the structural equation method, the minimum sample size should be 20 times higher than the number of subscales. There were 16 subscales in this case, so the minimum sample size required was 320. Assuming a 10% attrition rate, the final sample size estimated was 321 participants.

The Vice Chancellor of Nursing at the University of Medical Sciences was contacted to determine the appropriate number of healthcare professionals required in Ardabil City based on the population. The study included 140 nurses from Imam Khomeini Hospital (out of 560 individuals), 65 from Fatemi Hospital (out of 260 individuals), 40 from Alavi Hospital (out of 160 individuals), 35 from Imam Reza Hospital (out of 140 individuals), and 41 from Bu Ali Hospital (out of 164 individuals) using proportionate stratified sampling. Simple random sampling was used within each stratum. After obtaining the code of ethics, the researchers introduced themselves to the nursing offices of the teaching hospitals. Then, before sampling, they referred to different departments, introduced themselves to the nurses, and provided explanations about the research design and objectives and completing the questionnaires. Data were collected from January to March 2023.

### 3.5. Measures

#### 3.5.1. Social Demographics, Workplace Fun, Affective Commitment, Innovative Behavior and Innovation Support Questionnaire

The sociodemographic questionnaire included age, gender, marital status, education, work sector, experience, professional level, monthly income, shift type, and overtime.

#### 3.5.2. Workplace Fun

Workplace fun was measured using fourteen items adopted from Tews et al. [6]. This questionnaire has 14 items and three subgroups of socializing with colleagues, manager's support for fun, and attachment. Each item is rated on a five-point Likert scale, including completely disagree = 1, disagree = 2, neutral = 3, agree = 4, and completely agree = 5(6). The overall Cronbach's alpha coefficient of this questionnaire was reported to be 0.90 in the study of Tews et al. [6] and 0.86 in this study.

#### 3.5.3. Affective Commitment

The organizational commitment questionnaire was developed by Allen and Meyer [24]. It has three affective [1–8], continuous [9–16], and normative [17–24] dimensions. In this study, only the affective dimension of this questionnaire was used. Responses are scored on a five-point Likert scale, from completely disagree = 1, disagree to completely agree = 5; the lowest score is 8 and the highest score is 40. A higher score indicates more affective commitment. The Cronbach's alpha of the affective dimension was computed to be 0.85 in Allen and Mayer's study [24] and 0.73 in this study.

#### 3.5.4. Innovative Behavior Scale

This scale was prepared by Lukes and Stephan [3] by examining other innovative behavior scales. While reviewing the other scales, they deleted or modified some of the items in those scales. Innovative behavior scales include 23 questions and six dimensions. The six dimensions of innovative behavior include idea generation (3 items), idea search (3 items), idea communication (4 items), application (3 items), involving others (3 items), and overcoming obstacles (4 items). The overall mean score of the six subscales constitutes the overall score of the innovative behavior scale. The innovation output dimension consisting of three items is evaluated separately. Participants respond to questions on a five-point Likert scale, from strongly disagree = 1 to strongly agree = 5. An increase in the overall score of the innovative behavior scale and the subscale score of innovation output indicates an increase in innovative behavior and innovation output. The Cronbach's alpha values in the subscales of innovative behavior ranged from 0.60 to 0.88 in Lukes and Stephan's study [3] and from 0.79 to 0.96 in this study.

#### 3.5.5. Innovation Support Inventory

This scale was also developed by the same authors [3]. It includes 12 items and three dimensions, including managerial support, organizational support, and cultural support. The items are rated on a 5-point Likert scale, and increased scores of subscales indicate increased innovation support. Cronbach's alpha values of innovation support subscales varied from 0.77 to 0.82 in Lukes and Stephan's study [3] and from 0.91 to 0.93 in this study.

To use the workplace fun, innovative behavior, and innovation support questionnaires, after obtaining permission from the original designers, these instruments were subjected to forward-backward translation. First, they were translated into Persian by two expert translators who were blinded to each other. Then, both translations were merged into one by choosing the best words from each. Next, this selected Persian text was again translated into English by two translators who were fluent in English and did not know each other and the original text of the questionnaire. It was also ensured that the translated text matched the original text of the questionnaire. Then, before collecting the data, the content validity and face validity of the questionnaire were evaluated. For this purpose, the desired tool along with a list was provided to 14 nursing education experts and professors at Ardabil University of Medical Sciences, and they were asked to rate the items as necessary, useful but unnecessary, and unnecessary. Finally, the content validity indices of the workplace fun, innovative behavior, and innovation support questionnaires were calculated to be 0.89, 0.92, and 0.88, respectively. Then, to measure the reliability of the questionnaires, Cronbach's alpha coefficients were calculated for the questionnaires and their dimensions, whose values were above 0.79. These results showed that the questionnaires and their subscales had an acceptable level of reliability.

## 4. Data Analysis

This research implements variance-oriented partial least squares (PLSs) to conduct structural equation modeling. PLS is preferred due to its flexibility and reliability in handling complex models with smaller sample sizes. Two software, SmartPLS, and LISREL, adopt different approaches to evaluate the model fit. SmartPLS focuses on indicators related to the adequacy of the model in predicting dependent variables. Internal consistency, convergent validity, and predictive power are crucial indicators utilized to determine the model's goodness of fit.

The current research is of the type of structural equations in which the partial least squares method of the variance axis type is used. The difference between the variance-based method (partial least squares such as SmartPLS) and the covariance-based method (such as LISREL) is that this method does not require special distributional assumptions and is compatible with any number of samples, especially in more complex models with the number of variables is greater, even with a lower sample number, the results are still reliable. Also, this method has fewer limitations and is especially suitable in cases where predictive properties between variables are needed. In general, the power of this method is much higher than covariance-based modeling. This method has many validity indices that are used for composite structures. Also, at the alpha level of 0.01 and below, the statistical t-values higher than 1.96 are also considered significant.

In this research, the model of the relationships of workplace fun variables, affective commitment to innovation support, and innovative behavior as second-order and hierarchical constructs was tested in the form of a conceptual model through structural equation modeling, and the partial least squares method was used to test the measurement model. In testing the measurement model, convergent validity and average variance extracted (AVE) indices were used to assess validity [25]. To evaluate reliability, Cronbach's alpha (*α*) and composite reliability [26] were used. In the structural model section, the *R*^2^ and *Q*^2^ indices were used, the coefficient of determination or *R*^2^ for dependent variables shows the amount of explanation of the variable by the developed model [27, 28]. The *Q*^2^ index or Stone Geissler coefficient (sse/sso)-1, which is the quality index of the structural model, examines the predictive power of the structural model [28, 29]. The *f*^2^ index also shows the effect size of the independent variables. SPSS (version 14) and SmartPLS (2^nd^ version) were used for data analysis.

## 5. Ethical Considerations

This study was approved with the ethical code IR.ARUMS.REC.1401.153 by the Research Ethics Committee of Ardabil University of Medical Sciences. After obtaining the necessary permits, data collection was carried out from January to March 2023. Written informed consent was obtained from all participants. It was explained to the participants that the data would be kept anonymous and confidential. This survey was conducted on healthcare professionals who voluntarily agreed to participate in the study.

## 6. Results

### 6.1. Descriptive Statistics

In this research, 321 nurses in five hospitals in Ardabil received the set of questionnaires. All questionnaires were fully answered and analyzed.  Table 1 presents the descriptive indices, including mean, standard deviation, median, mode, skewness, and kurtosis related to each of the research variables.

### 6.2. Correlation Matrix

The correlation matrix, the relationship between constructs, and their significance are reported in Table 2(a–c).

The study focuses on the measurement model's reliability and validity using convergent validity and AVE. Convergent validity measures how well a hidden variable is understood by its items. Fornell and Larcker [25] established a criterion for the variance of this validity, where a value above 0.5 is deemed acceptable. For convergent validity, AVE values should be at least 0.5, and CR values should be at least 0.7. According to SmartPLS, all constructs in this study had a CR higher than 0.7, indicating convergent validity.

Divergent validity is checked by using a construct's square root (*e*), which should be higher than the correlation of that construct with other constructs [28]. This indicates that the correlation of that construct with its indicators is higher than its correlation with different constructs. Tables 2(a–c) presents the validity check results on the correlation matrix's diameter, showing the appropriate validity of the constructs.

### 6.3. Research Model Indicators

The study also evaluated the measurement model's reliability using Cronbach's alpha value of at least 0.7, which was considered acceptable. Another criterion called composite reliability was introduced by Werts et al. and is also used in the partial least square method. The values in Table 3(a–c) are more significant than 0.7, indicating that the measurement models possess good internal consistency. In addition, Section 6.3 [26] specifies the research model indicators. The reliability of the innovative behavior structure was 0.946 (Cronbach's alpha), and the convergent validity (AVE) and discriminant validity (AVE) were 0.495. The reliability of emotional commitment was 0.755, and the convergent and discriminant validity was 0.464. Lastly, the reliability for workplace fun was 0.982, and the convergent and discriminant validity was 0.451.

**Figure 2:**
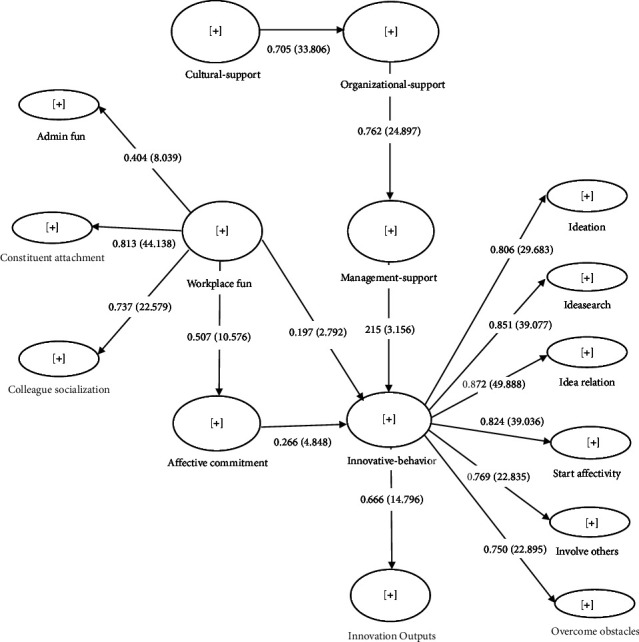
Factor-load coefficients significance of each model paths.

After verifying the validity of the constructs, it was found that the AVE index in three constructs, namely, innovative behavior (AVE = 0.495), affective commitment (AVE = 0.464), and workplace fun (AVE = 0.451), was below the recommended acceptable range suggested by Fornell and Larker. Specific questions with a lower factor load in each structure were removed from each scale to address this issue. Specifically, questions 1, 4, 13, and 14 were removed from the Workplace Fun scale, question 17 was removed from the Innovative Behavior scale, and question 8 was removed from the Affective Commitment scale. Following this, the AVE index was re-examined, and it was found to be 0.506, 0.543, and 0.503 for Innovative Behavior, Affective Commitment, and Workplace Fun, respectively. All three values were above 0.5 and considered acceptable. The model was then rerun, and the values of the indices, relationships between variables, and factor loadings were updated in Figure 2.

**Figure 3:**
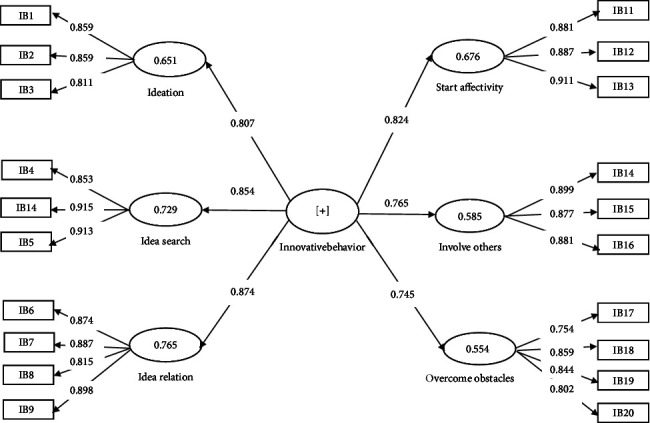
Factor-load coefficients significance of each innovative behavior's model paths.

**Figure 4:**
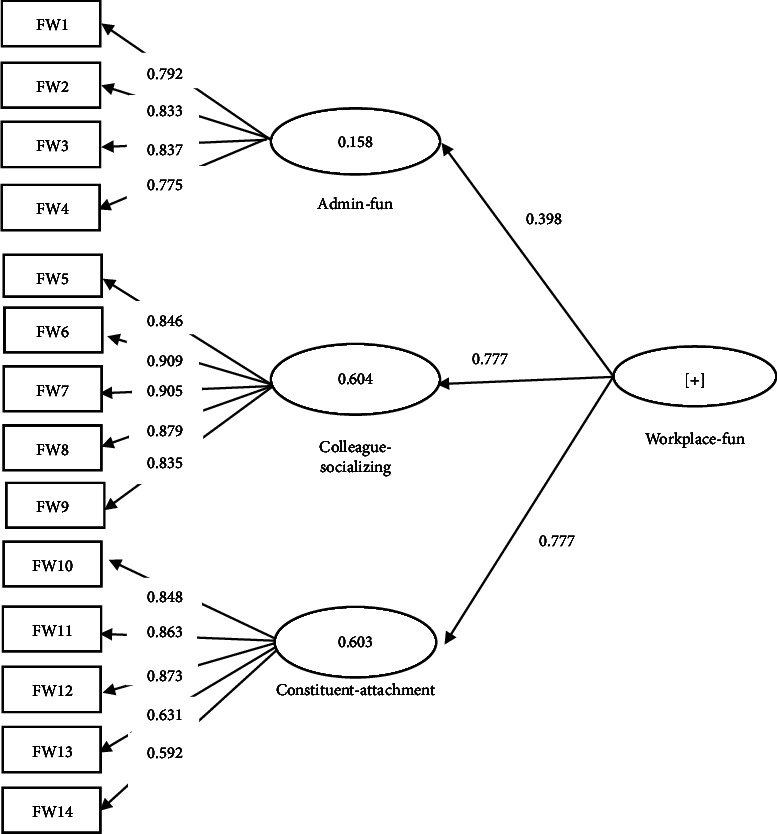
Factor-load coefficients significance of each work-place fun's model paths.

In addition, as per the referee's request, the hidden variables of the second order, i.e., workplace fun and innovative behavior, were run in separate models (Figures 3 and 4). The indices of convergent validity, divergent validity, and reliability of the subscales in these two models are presented and reported in Tables 2(b-c) and 3(b-c). All the reliability and validity indices for these two hidden variable subscales were within the acceptable range.

### 6.4. Factor Load Coefficients Significance of Each Model Paths

In examining the structural model, the coefficient of determination or *R*^2^ for dependent variables showed the degree of explanation of the variable by the developed model [27]. As shown in Table 3(a–c), the developed model was able to explain 48% of the variance of the innovative output variable, 35% of the variance of innovative behavior, 58% of the variance of managerial support and affective commitment, and 49% of the variance of organizational support, indicating that the structural model fit was moderately and strongly confirmed. The results of Table 3(a–c) show the *Q*^2^ index of 0.291 for innovative output, 156.0 for innovative behavior, 401.0 for managerial support, 200.0 for affective commitment, and 371.0 for organizational support, which indicates the moderate and high predictive power of the structural model. The *f*^2^ index also showed that the effect size of the independent variables was in the medium and high range. Finally, since the numbers on the main diameter of the Fornell and Larcker matrix (Table 2(a–c)) were more than the numbers below it, the validity of the research model was confirmed. The structural model of the research is reported as standard coefficients (PLS algorithm) and significant coefficients (bootstrapping) in Figure 2. Path coefficients (Beta) were used to determine the contribution of each of the predictor variables in explaining the variance of the criterion variable, and the significance of the path coefficients was also determined (Figure 2). According to the findings presented in Table 4 that shows factor loading of the studied variables, it can be inferred that workplace fun (WF) has a significant and positive impact on all the variables studied. In addition, innovative behavior (IB) also has a high impact on most of the variables. Lastly, affective commitment (AC) demonstrates moderate factor loadings on certain variables.

### 6.5. Direct paths Model

Considering the confirmation of the goodness of fit of the model, the model fitted to the data can be presented. Next, the significance of the direct paths was examined. The path of cultural support to organizational support was statistically significant (*β* = 0.705, *p* < 0.05). Assuming that other variables are constant, if cultural support increases by one unit, organizational support will increase by 0.705 standard deviations. Also, the paths of organizational support to managerial support (*β* = 0.762, *p* < 0.05), managerial support to innovative behavior (*β* = 0.192, *p* < 0.05), innovative behavior to innovation outputs (*β* = 0.678, *p* < 0.05), workplace fun to innovative behavior, (*β* = 0.278, *p* < 0.05), workplace fun to affective commitment (*β* = 0.467, *p* < 0.05), and affective commitment to innovative behavior (*β* = 0.248, *p* < 0.05) were statistically significant and positive (Table 5).

### 6.6. Indirect Paths Model

After examining the direct paths of the model (Table 6), the indirect paths were examined. For the path of cultural support to managerial support through organizational support, a significance level was reported (*p* < 0.001), indicating that cultural support has a significant effect on managerial support through the mediator variable organizational support. Assuming that other variables are constant, if cultural support increases by one unit, managerial support will increase by 0.537 standard deviations through organizational support. Also, the organizational support and managerial support variables were able to play a mediating role in the impact of cultural support on innovative behavior. Furthermore, for the path of cultural support to innovation outputs through the mediating variables innovative behavior, managerial support, and organizational support, a significant level of *p* value was reported (*p*=0.05) (Table 6), which shows that innovative behavior, organizational support, and managerial support could play a mediating role in the impact of cultural support on innovation outputs. Workplace fun had a significant effect on innovation outputs through the mediator variable innovative behavior. Finally, affective commitment and innovative behavior could play a mediating role in the impact of workplace fun on innovation outputs (Table 6).

## 7. Discussion

Rapid changes in global competition and the increasing demand for services from consumers make innovation essential for the survival of any organization. Due to the competitive environment of today's organizations, creating distinctive and continuous innovation by encouraging employees to generate ideas and implement ideas and innovations is one of the requirements for the success of organizations. There are effective factors for promoting the innovative behaviors of nurses in hospitals; the most effective ones are workplace fun, affective commitment, and innovative support. The main purpose of this study was to determine the relationship among workplace fun, affective commitment, innovation support, and innovative behavior. In other words, it aimed to find out how workplace fun, affective commitment, and innovation support influence innovative behavior.

Cultural support was related to organizational support and indirectly influenced managerial support through organizational support. This result was in line with the findings of Sonemz et al. [4]. Culture in organizations is the result of the relationships and interaction of the prejudices and assumptions of the founders of that organization, i.e., the sum of the common meanings and concepts between the members of the organization. One of the dimensions of culture in organizations is giving authority and support to employees to develop their capabilities, including innovation [30]. It can be argued that cultural support in organizations means the support of the members of the organizations for governing meanings. The organizational support theory assumes that organizational support fulfills the important socioaffective needs of employees at work, such as the need for affiliation or approval, which itself leads to self-enhancement processes in them [31]. Moreover, organizational support was associated with managerial support, which confirms the results of Sonmez et al.'s study [5]. Employees who receive more organizational support, in addition to organizational commitment, show more satisfaction, productivity, and creativity, which in turn can attract more attention and support from managers [32]. On the other hand, studies have shown that the more the employees receive better and more supportive leadership from their managers, the more organizational support they receive [33].

The results of the present study showed a relationship between managerial support and innovative behaviors, which was consistent with the study of Emiralioglu et al. [9] Managers and their support have direct and indirect effects on the culture and innovative behaviors of employees; they can exert this effect in different ways, including the effect of evaluation and reward system on innovation [34]. With the support of managers, employees can think more creatively about work-related issues and problem-solving [5]. In general, it can be said that managerial attitudes are significantly linked to innovation, and managers who encourage their employees to find new sources of knowledge inside and outside the organization experience a higher rate of innovation in their unit [34].

The results also indicated that cultural support significantly affected innovative behavior through the mediating role of managerial support and organizational support. This result was in line with the results of previous studies [3]. The results of these studies showed a relationship between managerial support and nurses' innovative behaviors. The results of other studies have revealed that the leadership style of nursing managers has a positive effect on behaviors [35, 36]. In this regard, the results of Sonmez et al.'s study [4] confirmed that nurses' innovative behaviors are influenced more by managerial innovation support, where cultural support affects organizational support and organizational support exerts an indirect effect on innovative behavior by influencing managerial support [4]. Organizations can improve innovation performance by motivating employees' innovative behavior and improving their wellbeing. They can also encourage innovation by developing innovation management systems, thereby creating a favorable environment for innovation and improving innovation performance [37].

The results also showed that cultural support significantly affected innovation outputs through the mediator variables managerial support, organizational support, and innovative behavior, which was consistent with the results of Lucas et al.'s study [3]. Through organizational support, managers and organizations can provide support or resources to facilitate employees' efforts to create positive changes, new ideas, or innovative behaviors [38]. At the organizational level, innovation performance refers to the successful implementation of original ideas. Zhang et al. [36] confirmed that organizational culture, organizational care, and social context affect employees' innovation performance. The results of these studies confirm that organizations can improve innovation performance by encouraging employees' innovative behavior and improving their wellbeing. They can also encourage innovation by developing innovation management systems, thereby creating a favorable environment for innovation and improving innovation performance. It can be said that innovative behavior is the most effective variable involved in nurses' innovation outputs. Hence, by improving and promoting innovative behaviors, innovative outputs can be directed in positive directions.

There was a relationship between innovative behaviors and innovative output, which was in agreement with the results of Sonmez et al. [4] and Emiralioglu et al. [9]. Although employees may show many innovative behaviors, these innovative behaviors do not always show themselves as innovative output, which can also affect employees due to the work environment [9]. Therefore, managers should always try to create an environment that can bring innovative behaviors closer to innovative output in employees.

Based on the results of this research, workplace fun directly and significantly predicted innovative behaviors. It also indirectly had a significant impact on innovation outputs through innovative behavior, which was consistent with the results of Jing [1] and Michel [15]. People feel happy when they participate in fun environments, which give them the necessary motivation to face challenges. Workplace fun can also create a kind of optimism and strengthen work values for a person [39]. Employees are more committed to their jobs when they have workplace fun because it can create an atmosphere that motivates them to invest their time and energy in work and stimulates their involvement in innovative work [9].

Furthermore, fun activities can effectively improve the working environment of employees and help them show more innovative behaviors [1]. Fun is always associated with joy and happiness and can create a kind of enthusiasm and mental flexibility for employees to be at work, which in turn leads to the creation of innovative behaviors in employees. The joy of workplace fun makes employees wants to do useful and productive work, use all their abilities to the fullest, and even enjoy helping others. Creating positive emotions maximizes people's energy for the development of physical, intellectual, and social resources and helps them make efforts to create new behaviors. Finally, improving nurses' innovative behaviors will increase the quality of services provided to patients and promote more appropriate innovative outputs.

The results indicated a significant and direct relationship between workplace fun and affective commitment and between affective commitment and innovative behaviors, which was consistent with the results of Jing's study [1]. Studies have reported that unpleasant and stressful factors in the workplace, such as violence and bullying, can have direct effects on reducing the affective commitment and mental health of employees [40]. Based on the affective events theory, work events can directly lead to affective reactions and contribute to judgment-oriented behavior. People's affective orientations affect their efforts to achieve occupational goals [13]. Accordingly, the results of the present study can be because the presence of workplace fun creates this feeling in employees that they are in a suitable environment and feel emotionally united with the organization, which is more important given the effects of affective commitment on healthcare providers and patients. People who have a high level of affective commitment in the organization are more productive than others and use it to creatively and innovatively solve workplace problems. Their innovative work behavior also appears as a high social exchange relationship in these people, and these people enjoy higher affective commitment [40].

In addition, nurses' affective commitment and innovative behavior had a mediating effect between workplace fun and innovation output. In the hospital work environment, nurses participate in fun activities, enjoy the fun environment, and practice fun socializing, which can stimulate their attachment to the hospital from a personal affective perspective. This positive feeling can make nurses develop innovative behaviors for hospital performance [5]. The main goal of innovative behavior is to achieve innovative outputs. Innovation outputs are obtained when a product or method in an organization is developed by implementing new ideas or when new ideas are used by modifying existing products or methods in the organization [5].

## 8. Limitations

This study has a few limitations that need to be taken into account. First, the study's design was cross-sectional, meaning the findings cannot be generalized to other health organizations. Therefore, future studies should adopt longitudinal designs for better generalizability. Second, self-reporting was used to collect data for this study. However, self-reporting to evaluate innovative behavior and innovation outputs may lead to biased results, so qualitative and interventional studies are required. Lastly, this study only focused on nurses but did not consider doctors and administrators working in the same hospitals, which limits generalizability. The authors faced limitations in accessing newer versions of SmartPLS software in Iran, so they had to use version 2, which can affect statistical analysis results. Future studies should consider using the latest version to avoid software version-related limitations. In addition, considering other hospital workers in future studies can provide a deeper insight into the relationship between variables.

## 9. Implications for Nursing Management

By providing organizational and managerial support for nurses' innovative behaviors, managers should take measures that promote workplace fun and affective commitment to improve nurses' innovative outputs by encouraging their innovative behaviors.

## 10. Conclusion

Workplace fun, cultural support, managerial support, and organizational support had a positive and significant effect on nurses' innovative behaviors. In addition, workplace fun could indirectly cause innovative output behaviors in nurses through innovative behavior. Also, workplace fun using the mediating role of affective commitment and innovative behavior had a positive and significant effect on nurses' innovation output. Accordingly, supervisors and managers can adopt the organizational and managerial support approach to increase nurses' innovative behaviors. Workplace fun as a practical motivational approach can have a positive effect on nurses' attitudes, thereby enhancing their affective commitment and innovative behaviors. With the increase in innovative behaviors, the nurses' innovative outputs will also increase. For managers and practitioners dealing with the issue of innovation, a conscious understanding of innovative behaviors and innovation support factors may help them to focus on the strengths of innovation, reduce weaknesses, and manage their innovation more efficiently.

## Figures and Tables

**Table 1:** Descriptive statistics of research variables.

Variable	Elongation	Crookedness	SD	Mod	Middle	Mean
Innovation outputs	0.465	−0.107	0.762	3.00	3.33	3.307
Cultural support	−0.216	0.193	0.918	3.00	2.60	2.505
Innovative behavior	1.263	−0.584	0.627	4.00	3.59	3.515
Managerial support	−0.684	−0.083	0.957	3.00	3.00	2.802
Affective commitment	1.00	0.342	0.610	3.00	3.00	3.083
Organizational support	−0.610	0.202	1.010	3.00	2.66	2.526
Workplace fun	0.611	−0.154	0.627	3.00	3.00	2.951

**Table 2:** (a) Fornell and Larcker matrix and extracted root mean variance of research variables. (b) Fornell and Larcker matrix and extracted root mean variance of innovative behavior subscales. (c) Fornell and Larcker matrix and extracted root mean variance of workplace fun subscales.

(a)							
Variable	1	2	3	4	5	6	7

1	0.799^*∗∗∗*^						
2	0.361^*∗∗∗*^	0.837^*∗∗∗*^					
3	0.682^*∗∗∗*^	0.303^*∗∗*^	0.709^*∗∗∗*^				
4	0.494^*∗∗∗*^	0.585^*∗∗∗*^	0.418^*∗∗∗*^	0.857^*∗∗∗*^			
5	0.411^*∗∗∗*^	0.366^*∗∗∗*^	0.456^*∗∗∗*^	0.345^*∗∗∗*^	0.736^*∗∗∗*^		
6	0.434^*∗∗∗*^	0.605^*∗∗∗*^	0.309^*∗∗∗*^	0.762^*∗∗∗*^	0.345^*∗∗∗*^	0.888^*∗∗∗*^	
7	0.358^*∗∗∗*^	0.271^*∗∗*^	0.511^*∗∗∗*^	0.424^*∗∗∗*^	0.487^*∗∗∗*^	0.316^*∗∗∗*^	0.709^*∗∗∗*^

(b)							
Variable	1	2	3	4	5	6	

1	0.885^*∗∗∗*^						
2	0.622^*∗∗∗*^	0.816^*∗∗∗*^					
3	0.553^*∗∗∗*^	0.561^*∗∗∗*^	0.893^*∗∗∗*^				
4	0.554^*∗∗∗*^	0.505^*∗∗∗*^	0.713^*∗∗∗*^	0.869^*∗∗∗*^			
5	0.575^*∗∗∗*^	0.508^*∗∗∗*^	0.604^*∗∗∗*^	0.742^*∗∗∗*^	0.894^*∗∗∗*^		
6	0.507^*∗∗∗*^	0.574^*∗∗∗*^	0.575^*∗∗∗*^	0.633^*∗∗∗*^	0.687^*∗∗∗*^	0.844^*∗∗∗*^	

(c)							
Variable		Colleague socializing		Constituent attachment		Admin fun	

Colleague socializing		0.875^*∗∗∗*^					
Constituent attachment		0.272^*∗∗*^		0.771^*∗∗∗*^			
Admin fun		0.240^*∗∗*^		0.390^*∗∗*^		0.810^*∗∗∗*^	

Noticeable: *P* < 0.001^*∗∗∗*^ and *P* < 0.01^*∗*^. (a) 1 = innovation outputs; 2 = cultural support; 3 = innovative behavior; 4 = managerial support; 5 = affective commitment; 6 = organizational support; 7 = workplace fun. (b) 1 = involve others; 2 = overcome obstacles; 3 = start affectivity; 4 = idea relation; 5 = idea search; 6 = ideation. When interpreting the Fornell and Larcker matrix, compare the values. The bold values indicate the square roots of the Average Variance Extracted (AVE) values. Ensure that the values along the main diagonal are greater than the other values in the corresponding column, as this confirms the structure's validity.

**Table 3:** (a) Research model indicators and their acceptable values. (b) Model indicators and their acceptable values. (c) Workplace fun model indicators and their acceptable values.

(a)						
Variable	AVE <0.5	CR >0.7	(*α*) Cronbach's alpha >0.7	ƒ^2^	*R* ^2^ Weak: 0.19 Medium: 0.33 Strong: 0.67	*Q* ^2^ Weak: 0.02 Medium: 0.15 Strong; 0.35

Innovation outputs	0.638	0.841	0.717		0.465	0.279
Cultural support	0.700	0.921	0.898	0.869		
Innovative behavior	0.506	0.950	0.945		0.311	0.140
Managerial support	0.735	0.917	0.879		0.581	0.401
Affective commitment	0.541	0.847	0.766		0.272	0.136
Organizational support	0.788	0.918	0.866		0.497	0.371
Workplace fun	0.503	0.856	0.811	0.373		

(b)						
Variable	AVE <0.5	CR >0.7	(*α*) Cronbach's alpha >0.7	*R* ^2^ Weak: 0.19 Medium: 0.33 Strong: 0.67		*Q* ^2^ Weak: 0.02 Medium: 0.15 Strong: 0.35

Involve-others	0.784	0.916	0.862	0.585		0.434
Overcome obstacles	0.666	0.888	0.832	0.554		0.345
Start affectivity	0.797	0.922	0.873	0.678		0.510
Idea relation	0.755	0.925	0.892	0.765		0.542
Idea search	0.800	0.923	0.874	0.729		0.551
Ideation	0.712	0.881	0.797	0.651		0.439

(c)						
Variable	AVE <0.5	CR >0.7	(*α*) Cronbach's alpha >0.7	*R* ^2^ Weak: 0.19 Medium: 0.33 Strong: 0.67		*Q* ^2^ Weak: 0.02 Medium: 0.15 Strong: 0.35

Colleague socializing	0.766	0.942	0.924	0.604		0.427
Constituent attachment	0.595	0.887	0.831	0.603		0.326
Admin fun	0.655	0.884	0.827	0.258		0.193

**Table 4:** Factor loading of the studied variables.

Question	Load factors	*t*-value	Question	Load factors	*t*-value	Question	Load factors	*t*-value
FW1	0.792	9.465	IB6	0.913	73.917	IB25	0.875	55.331
FW2	0.833	9.968	IB7	0.887	54.721	IB26	0.0899	68.596
FW3	0.837	11.863	IB8	0.816	22.849	IB27	0.838	34.778
FW4	0.775	12.379	IB9	0.898	59.343	IB28	0.867	43.337
FW5	0.846	13.718	IB10	0.874	47.417	IB29	0.908	58.616
FW6	0.909	13.643	IB11	0.881	54.211	IB30	0.888	53.295
FW7	0.905	13.669	IB12	0.887	53.458	IB31	0.762	37.492
FW8	0.879	13.332	IB13	0.911	84.478	IB32	0.876	52.443
FW9	0.835	10.594	IB14	0.899	64.749	IB33	0.874	39.883
FW10	0.848	15.334	IB15	0.877	31.495	IB34	0.868	37.108
FW11	0.863	14.940	IB16	0.881	48.696	IB35	0.799	23.162
FW12	0.873	23.502	IB17	0.754	24.146	AC1	0.852	39.849
FW13	0.631	9.384	IB18	0.859	47.476	AC2	0.793	28.260
FW14	0.592	7.773	IB19	0.844	38.506	AC3	0.777	23.971
IB1	0.859	51.088	IB20	0.802	28.994	AC4	0.486	3.252
IB2	0.859	49.411	IB21	0.867	52.570	AC5	0.451	2.601
IB3	0.811	34.398	IB22	0.729	16.280	AC6	0.467	2.800
IB4	0.853	37.671	IB23	0.795	26.408	AC7	0.757	17.27
IB5	0.915	59.867	IB24	0.814	29.133	AC8	0.434	2.85

Noticeable: WF = workplace fun; IB = innovative behavior; AC = affective commitment.

**Table 5:** Direct paths model.

Direct paths	Significance level	*T*-test	Coefficient path	Result
CS ⟶ OS	*p* < 0.001	33.806	0.705	Confirmation
OS ⟶ MS	*p* < 0.001	24.897	0.762	Confirmation
MS ⟶ IB	0.005	3.156	0.215	Confirmation
IB ⟶ IO	*p* < 0.001	14.796	0.666	Confirmation
WF ⟶ IB	*p* < 0.001	2.792	0.197	Confirmation
WF ⟶ AC	*p* < 0.001	10.576	0.507	Confirmation
AC ⟶ IB	*p* < 0.001	4.848	0.266	Confirmation

Noticeable: CS = cultural support; OS = organizational support; MS = managerial support; IB = innovative behavior; IO = innovation outputs; WF = workplace fun; AC = affective commitment.

**Table 6:** Indirect paths model.

Indirect paths	Coefficient path	Sobel test	Significance-level	Result
CS ⟶ OS ⟶ MS	0.537	18.129	*p* < 0.001	Confirmation
CS ⟶ OS ⟶ MS ⟶ IB	0.116	3.039	0.002	Confirmation
CS ⟶ OS ⟶ MS ⟶ IB ⟶ IO	0.077	2.805	0.005	Confirmation
WF ⟶ IB ⟶ IO	0.131	2.792	0.005	Confirmation
WF ⟶ AC ⟶ IB ⟶ IO	0.090	4.030	*p* < 0.001	Confirmation

Noticeable: CS = cultural support; OS = organizational support; MS = managerial support; IB = innovative behavior; IO = innovation outputs; WF = workplace fun; AC = affective commitment.

## Data Availability

The data used to support the findings of this study are available from the corresponding author upon reasonable request.
